# Serotonin transporter functional polymorphisms potentially increase risk of schizophrenia separately and as a haplotype

**DOI:** 10.1038/s41598-022-05206-x

**Published:** 2022-01-25

**Authors:** Rana Ghamari, Fatemeh Yazarlou, Zahra Khosravizadeh, Atefeh Moradkhani, Elaheh Abdollahi, Fatemeh Alizadeh

**Affiliations:** 1 Department of Genetics, Faculty of Biology, Kharazmi University, Tehran, Iran; 2 Department of Medical Genetics, School of Medicine, Tehran University of Medical Sciences, Tehran, Iran; 3 Clinical Research Development Unit, Infertility treatment clinic, Amiralmomenin Hospital, Arak University of Medical Sciences, Arak, Iran; 4 Department of Biology, Faculty of Science, Zanjan Branch, Islamic Azad University, Zanjan, Iran; 5 Department of Medical Genetics, Faculty of Medical Sciences, Tarbiat Modares University, Tehran, Iran; 6 Department of Genomic Psychiatry and Behavioral Genomics (DGPBG), Roozbeh Hospital, School of Medicine, Tehran University of Medical Sciences, Tehran, Iran

**Keywords:** Genetics, Neuroscience

## Abstract

Schizophrenia is a severe, disabling psychiatric disorder with unclear etiology. Family-based, twins, and adoption studies have shown that genetic factors have major contributions in schizophrenia occurrence. Until now, many studies have discovered the association of schizophrenia and its comorbid symptoms with functional polymorphisms that lie within serotonin reuptake pathway genes. Here, we aimed to investigate the association of three variable number tandem repeats (VNTR) functional polymorphisms in *MAOA* and *SLC6A4* with schizophrenia in the Iranian population. Two hundred and forty-one subjects with schizophrenia and three hundred and seventy age and sex-matched healthy controls were genotyped for *MAOA* promoter uVNTR, 5-HTTLPR, and STin2 polymorphisms. Genotyping was performed by polymerase chain reaction (PCR) with locus-specific primers and running the PCR product on agarose 2.5% gel electrophoresis. Finally, the statistical inference was performed using R programming language and Haploview software. *MAOA* promoter uVNTR analysis of allele frequency showed no differences between schizophrenia subjects and healthy controls in both males and females and no significant differences were observed between female cases and female controls in *MAOA* promoter uVNTR 4 repeat frequency. Also, there were no differences between Schizophrenia and healthy control groups in 5-HTTLPR allele and genotype frequency but, 5-HTTLPR S allele carriers are significantly more frequent among cases. In addition, STin2.12 repeats were significantly more frequent among schizophrenia patients. Genotype comparison suggested that 5-HTTLPR S allele and STin2.12 repeat carriers were significantly more frequent among schizophrenia cases and being STin2.12 repeat carrier significantly increase the risk of schizophrenia occurrence. Besides, analysis of haplotype showed stronger linkage disequilibrium between 5-HTTLPR and STin2 haplotype block in cases than controls. These results suggest that *SLC6A4* functional polymorphisms potentially could play a possible role as risk factors for the incidence of schizophrenia.

## Introduction

Schizophrenia (SCZ) is one of the five major psychiatric disorders with an approximate prevalence of 1% worldwide^1^. As a multifactorial disease, the exact etiology of SCZ is still unknown. However, family-based, twins, and adoption studies suggest the contribution of 46%-80% of genetic factors in the incidence and pathogenesis of SCZ^2^. Even though multiple genes and pathways take a part in the incidence and severity of SCZ, the involvement of monoamine neurotransmitters is one of the most probable theories for decades^3^.

Serotonin (5-HT) is a neurotransmitter responsible for setting various functions such as emotional features, anxiety traits, aggression, and Etc^4^. Earlier, Several studies have given shreds of evidence to show the substantial role of the 5-HT and serotonergic pathway in the pathophysiology of SCZ and other psychiatric disorders^5^. Serotonin transporter (5-HTT), encoded by the *SLC6A4* gene (location:17q11.1–q12), is known as one of the major regulators of 5-HT via reuptake serotonin from synaptic clefts^6–8^. Following the reuptake process, 5-HT is degraded in presynaptic neurons by monoamine oxidase enzymes including monoamine oxidase A (MAOA) enzyme, which, encoded by the *MAOA* gene at Xp11.3 locus^9,10^. Thus far, many studies have revealed that changes in mRNA and protein levels of 5-HTT and MAOA are associated with behavioral traits and psychiatric disorders including SCZ^11–13^.

On the other aspect, it is well established that variable number tandem repeats (VNTRs) functional polymorphisms are groups of polymorphisms that could affect on expression of genes through changing the number of repeats^14^. Recently, Rasekh et al. have found that about 180 different genes expression correlated with their proximal VNTRs sequences^15^. This Phenomenon clearly can be seen in both 5-HTT and MAOA. Serotonin transporter linked polymorphic region (5-HTTLPR) is a 44 bp element VNTR functional polymorphism located in the promoter region of *SLC6A4* and has been frequently studied in psychiatric related conditions in humans including bipolar disorder, SCZ, Obsessive–Compulsive Disorder, and evaluation in response to medication therapy in depression^16–19^. Besides, in macaques, 5-HTTLPR has shown a significant correlation with aggression-related behavior^20^. L and S alleles are 5-HTTLPR major alleles which S allele results in lower expression and the L allele cause higher transcriptional activity^21,22^. Up to now, several studies investigate the role of different genotypes of 5-HTTLPR in psychosis. Although the latest Meta-Analysis showed no association between 5-HTTLPR and SCZ, a few populations discovered 5-HTTLPR association with SCZ^23^. For example, the association of the 5-HTTLPR “L” allele with SCZ in the Indian population was the earliest study that found a significant relation between SCZ and 5-HTTLPR^24^. In 2016, Golimbet et al. showed a higher frequency of the SS genotype in affective psychosis compared to healthy individuals. In addition, in the same year, Xu-Xiu et al. found that S/L genotype is a SCZ risk factor in Han Chinese population^25,26^. It is worth mentioning that recent articles have focused on the association of 5-HTTLPR with SCZ comorbid symptoms, but, 5-HTTLPR association with SCZ risk in many populations remained as an enigma.

Another VNTR functional polymorphism of 5-HTT, the serotonin transporter intron 2 VNTR (STin2) is a 17 bp VNTR element usually existing in 9, 10, and 12 repeats and effects on 5-HTT transcription through binding to transcription factor Y-box binding protein 1 (YB-1)^27–29^. Pieces of evidence have shown that STin2.12 repeats elevate the expression of 5-HTT compared to STin2.9 and STin2.10 variants^30^. Several studies support the idea of STin2 association with psychiatric disorders such as obsessive–compulsive disorder (OCD), SCZ and personality traits including Neuroticism and Harm Avoidance^28,31,32^. Unlike the 5-HTTLPR, there are much evidence for a strong association between STin2.12 repeats and SCZ in many populations including Caucasians, Indians, East Asians, etc.^23^.

Moreover, *MAOA* functional polymorphism (uVNTR) is a 30 bp sequence located in the MAOA promoter region and the number of copies varies from 2 to 5 which the 4 repeats increase the expression of *MAOA* up to 10 times^9^. Association of MAOA uVNTR with aggressive behaviors in SCZ, antisocial problems, and impulsivity are a few examples of MAOA expression effect on behavioral traits^33–36^. Knockout uVNTR in semi-haploid HAP1 cell lines appears the role of uVNTR in *MAOA* expression alteration^37^. Association of uVNTR with SCZ in Mexican, Han Chinese, and Croatian populations are examples of MAOA VNTR relation with risk of SCZ in different populations^38–40^.

Although *MAOA* and *SLC6A4* are two parts of the same pathway and the Possibility of 5-HTTLPR and STin2 involvement in physical linkage, few studies have been focused on haplotype analysis of these two genes variants. For instance, in 2008, V. Kazantseva et al. illustrated 5-HTTLPR S and STin2.12 haplotype (S-12) carriers show higher anxiety-related traits such as harm avoidance (D′ = 0.36, r^2^ = 0.12)^32^. In addition, Guhathakurta et al. revealed the linkage disequilibrium (LD) of STin2.10 with T allele of 5-HTT-3′UTR-SNP in autistic individuals of the Indian population (D′ = 0.82, r^2^ = 0.34, LOD = 6.63)^41^.

Taken together aforementioned, until now, no study has investigated the *MAOA* uVNTR, 5-HTTLPR, and STin2 LD analysis and allelic interaction in schizophrenic patients. Thus, the purpose of this study was to evaluate the association of *MAOA* uVNTR, 5-HTTLPR, and STin2 with SCZ in the Iranian population and investigation of LD and allele interaction in mentioned functional polymorphisms.

## Material and methods

After a full explanation of the possible consequences of participating in this study, Informed consent was obtained from the participants and their legal guardians. The whole process of this study has been carried out according to the criteria of the Helsinki Declaration and approved by the Iran National Committee for Ethics in Biomedical Research and the Ethics Committee of Tehran University of Medical Sciences, Tehran, Iran (IR.TUMS.MEDICINE.REC.1399.145).

### Participants

Two hundred and forty-one schizophrenic patients and three hundred and seventy healthy individuals participated in the study. SCZ group consists of 50 females and 191 males (age mean = 38.59 ± 11.05) diagnosed by two independent expert adult psychiatrists according to the diagnosis criteria of Diagnostic and Statistical Manual of Mental Disorders, 5th Edition (DSM-5) in Roozbeh psychiatry hospital. All cases had no comorbidity with other mental illnesses and metabolic or immune system related disorders. Also, healthy controls consist of 76 females and 273 males (age mean = 37.38 ± 9.86) who became acquainted with this study through advertising and participated voluntarily. None of the healthy subjects had neither familial nor personal histories of mental, immunological, or metabolic disorders. Both schizophrenic patients and healthy subjects had Iranian ancestry and were unrelated either between groups or within groups.

### Genotyping

5 ml of peripheral blood was collected from all participants. Genomic DNA was extracted from whole blood using the well-established salting-out protocol in the Department of Genomic Psychiatry and Behavioral Genomics (DGPBG) Roozbeh Psychiatry Hospital^42^. DNA Concentration and contamination were evaluated respectively by UV-spectroscopy and optical density ratios. Besides, qualitative assessment of DNA checked by running the final extraction product on 1% agarose gel electrophoresis. Genotyping of MAOA uVNTR, 5-HTTLPR, and STin2 was performed by polymerase chain reaction (PCR) with locus-specific primers. Afterward, following the PCR step, the PCR product ran on 2.5% agarose gel electrophoresis, and finally, the results were confirmed by running the PCR product on 8% polyacrylamide gel electrophoresis (PAGE). Primers were designed according to the previous studies^43–45^ and using primer-BLAST web server for every functional polymorphism which is shown in Table 1.

**Table 1:** Forward (F) and reverse (R) primers for functional polymorphisms uVNTR, 5-HTTLPR and STin2.

Polymorphism	F	R
uVNTR	CCCAGCGTGCTCCAGAAA	GGACCTGGGCAGTTGTGC
5-HTTLPR	GGCGTTGCCGCTCTGAATGC	GAGGGACTGAGCTGGACAACCAC
STin2	TGGATTTCCTTCTCTCAGTGATTGG	TCATGTTCCTAGTCTTACGCCAGTG

### Statistical analysis

All statistical analysis performed in R version 4.0.4 (R Core Team, 2021), ggplot2(H. Wickham. ggplot2: Elegant Graphics for Data Analysis. Springer-Verlag New York, 2016.) and dplyr (Hadley Wickham, Romain François, Lionel Henry and Kirill Müller (2020). dplyr: A Grammar of Data Manipulation. R package version 1.0.2. https://CRAN.R-project.org/package=dplyr) packages. Haploview software version 4.2 applied for analysis of linkage disequilibrium using expectation–maximization (EM) algorithm^46^. The normality of ages in participants was checked using the Shapiro–Wilk test. A Two-sided Mann–Whitney test was used for comparison of mean ages between cases and controls in significance level of 95% (alpha = 0.05). Sex frequency comparison between two groups, deviation from Hardy–Weinberg equilibrium (HWE) for cases and controls, comparison of allele frequency between cases and controls, the association of all three functional polymorphisms with SCZ, haplotype differences between both groups, and genotypic interaction calculated using two-tailed Pearson's chi-squared test in significance level of 95% (alpha = 0.05). A Two-tailed Fisher exact test was used for Odds ratio calculation. At last, logistic regression was performed to prediction of SCZ risk with functional polymorphisms and 5-HTTLPR and STin2 interaction. Also, Bonferroni correction was used for adjusting the *p* value obtained from multiple comparison tests. It should be noticed that as a rare condition, carriers of STin2.9 repeats were excluded from the analysis of STin2 alleles and genotypes. In addition, as an X-linked variant, MAOA uVNTR allele analysis was carried- out separately in males, and females and genotype association were executed only in females.

## Results

### Demographic results

Mann–Whitney and Pearson's chi-squared tests respectively showed there were no significant differences between cases and controls in age (W = 39,066, *p* value = 0.17) and sex (χ-squared = 0.04, df = 1, *p* value_chi-squared_ = 0.84) with 95% significance level. The demographic results have shown briefly in Table 2.

**Table 2:** Demographic data.

	Male	Female	Mean age	SD
Case	191	50	38.59	11.05
Control	273	76	37.38	9.86
*P* value	0.84		0.17	

### Allele frequency analysis

Table 3 shows allele frequency of *MAOA* uVNTR, 5-HTTLPR and STin2 in both subjects and healthy individuals. Applying Pearson's chi-squared test revealed there were no significant differences in allele frequency of *MAOA* uVNTR neither in males nor females (males: χ-squared = 1.38, df = 1, *p* value_chi-squared_ = 0.70/females: χ-squared = 4.33, df = 1, *p* value_chi-squared_ = 0.22) and 5-HTTLPR (χ-squared = 2.85, df = 1, *p* value_chi-squared_ = 0.10) between cases and controls. However, STin2 allele frequency showed significant differences between cases and control subjects (χ-squared = 6.72, df = 1, *p* value_chi-squared_ = 0.09*10^−1^) and STin2.12 is more frequent among Schizophrenic individuals (Odds ratio = 1.42, *p* value_Fisher’s exact_ = 0.08*10^−1^).

**Table 3:** Allele frequency of MAOA uVNTR, 5-HTTLPR and STin2.

Allele	MAOA uVNTR				5-HTTLPR		STin2	
	2 repeats	3 repeats	4 repeats	5 repeats	L	S	10 repeats	12 repeats
**Case**								
Male	2 (1%)	70 (41%)	91 (54%)	5 (4%)	217 (45%)	265 (55%)	118 (24%)	361 (76%)
Female	3 (3%)	40 (4%)	51 (51%)	6 (6%)				
**Control**								
Male	4 (1%)	96 (36%)	157 (59%)	9 (4%)	371 (50%)	369 (50%)	234 (32%)	504 (68%)
Female	2 (1%)	71 (44%)	85 (52%)	3 (3%)				
**Total**								
Male	6 (1%)	166 (38%)	248 (57%)	14 (4%)	588 (48%)	634 (52%)	352 (29%)	865 (71%)
Female	5 (2%)	111 (43%)	136 (52%)	9 (3%)				
***P* value**								
Male	0.70				0.09		0.09*10^−1^	
Female	0.22							

### Association analysis

For *MAOA* uVNTR, there were significant differences in genotypic frequency distribution between cases and controls (χ-squared = 11.40, df = 3, *p* value_chi-squared_ = 9.75*10^−3^) with alpha of 95%. 5-HTTLPR presented almost significant differences between schizophrenic patients and healthy controls but, it couldn’t pass the 95% significance level (χ-squared = 5.46, df = 2, *p* value_chi-squared_ = 0.06). About STin2 we observed significant association (χ-squared = 15.64, df = 2, *p* value_chi-squared_ = 0.03*10^−2^) between SCZ and STin2.12. After genotypic association analysis, a fitting genetic model was applied for 5-HTTLPR and STin2. “S” allele in 5-HTTLPR and 12 repeats in STin2 are supposed as risk alleles according to the previous studies^47,48^. Therefore, Pearson’s Chi-squared test demonstrated that carrying “S” allele (SS and SL) was significantly associated with SCZ (χ-squared = 4.94, df = 1, *p* value = 0.02) and significantly more frequent among cases (Odds ratio = 1.60, *p* value_Fisher’s exact_ = 0.02). Besides, the results discriminated that being STin2.12 carrier, was significantly associated with SCZ (χ-squared = 15.25, df = 1, *p* value_chi-squared_ = 9.38*10^−5^). Also, Fisher's Exact Test represented that carrying STin2.12 was more frequent between schizophrenic patients (Odds ratio = 3.85, *p* value_Fisher’s exact_ = 3.27*10^−5^). Finally, Pearson Chi-squared revealed almost significant association between lack of MAOA uVNTR 4 repeats and SCZ (χ-squared = 3.53, df = 1, *p* value_chi-squared_ = 0.06). Genotypic frequencies and genetic models have displayed in Tables 4 and 5.

**Table 4:** Genotypic frequency of MAOA uVNTR, 5-HTTLPR and STin2.

Genotype	MAOA uVNTR				5-HTTLPR			STin2		
	2/4	3/3	3/4	4/4	LL	LS	SS	10/10	10/12	12/12
Case (females)	1 (2%)	16 (35%)	8 (17%)	21 (46%)	46 (19%)	125 (51%)	70 (30%)	10 (4%)	98 (43%)	130 (52%)
Total	46				241			238		
Control (females)	2 (3%)	13 (4%)	34 (46%)	24 (47%)	101 (27%)	169 (45%)	100 (28%)	52 (14%)	130 (35%)	187 (51%)
Total	73				370			369		
*P* value	9.75*10^−3^				0.06			0.03*10^−2^

**Table 5:** Genetic model of 5-HTTLPR and STin2.

Model	MAOA uVNTR		5-HTTLPR		STin2	
	2,3,4/4	2,3/2,3	LL	S-	10/10	12/ (10,12)
Case	30(65%)	16(35%)	46 (19%)	195 (81%)	10 (4%)	228 (96%)
Total	46		241		238	
Control	60(82%)	13(18%)	101 (27%)	269 (73%)	52 (14%)	317 (86%)
Total	73		370		369	
*P* value	0.06		0.02		9.38*10^−5^	

S-: SS and SL.

### 5-HTTLPR & STin2 interaction and haplotype analysis

**Figure 1:**
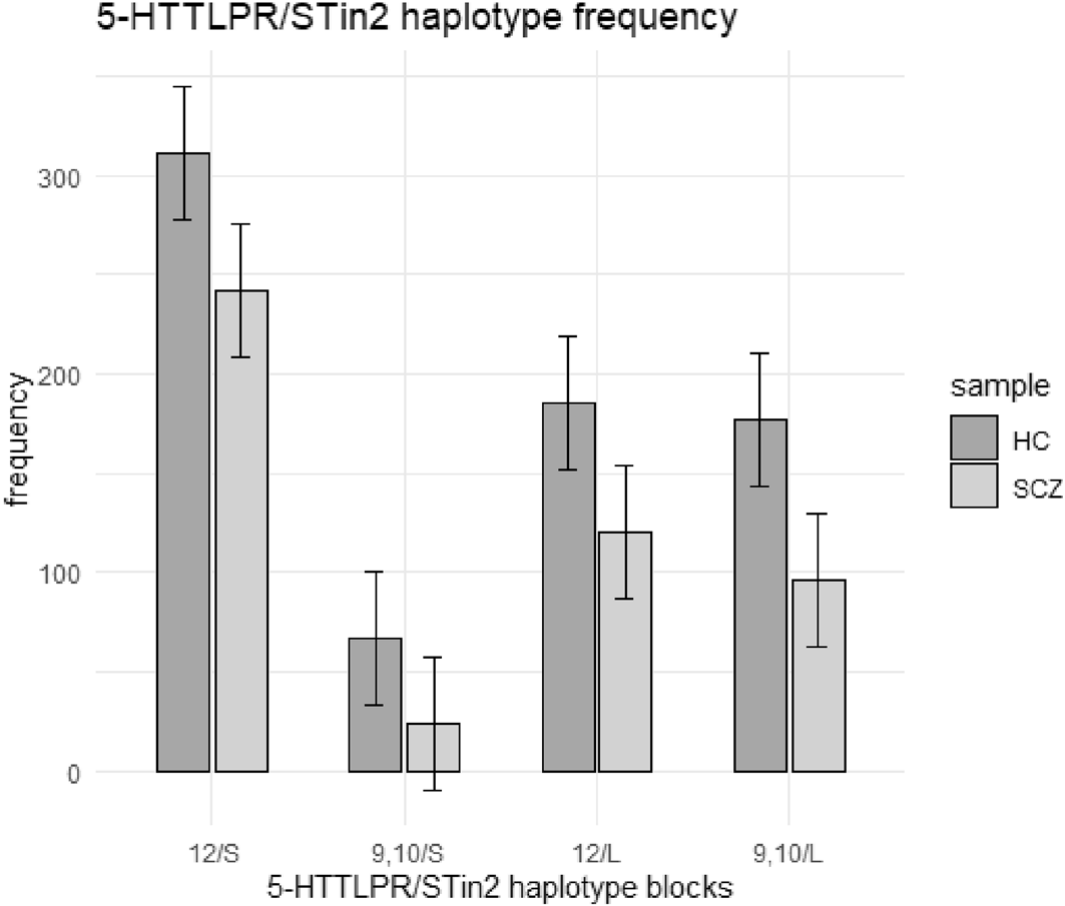
Haplotype frequency in schizophrenic patients and healthy controls.

Pearson's Chi-squared test which was followed by Fisher's Exact Test illustrated that either in cases/controls separately or in total population, there was significant association between being “S” carrier allele and STin2.12 repeat carrier (cases : χ-squared = 4.53, df = 1, *p* value_chi-squared_ = 0.03, Odds ratio = 4.60, *p* value_Fisher’s exact_ = 0.02; controls : χ-squared = 36.08, df = 1, *p* value_chi-squared_ = 1.90*10^−9^, Odds ratio = 600.46*10^−2^, *p* value_Fisher’s exact_ = 6.70*10^−9^; total: χ-squared = 48.35, df = 1, *p* value_chi-squared_ = 3.55*10^−12^, Odds ratio = 6.10, *p* value_Fisher’s exact_ = 6.83*10^−11^). Moreover, *p* value extracted from general linear model showed significant interaction between STin2.12 and 5-HTTLPR “S” allele after exclusion of confounding factors (intercept = 0.78, *p* value_adjusted_ = 4.80*10^−11^). Analysis of haplotype revealed that there is a significant difference in haplotype frequency between schizophrenic subjects and healthy controls (χ-squared = 12.91, df = 3, *p* value_chi-squared_ = 4.81*10^−3^). Further analysis revealed that S-12 haplotype significantly associated with SCZ (χ-squared = 7.55, df = 1, *p* value_chi-squared_ = 5.97*10^−3^, *p* value_Fisher’s exact_ = 5.70*10^−3^, Odds ratio = 1.40), whereas, S-10 haplotype were significantly more frequent among healthy individuals (χ-squared = 6.45, df = 1, *p* value_chi-squared_ = 0.01, *p* value_Fisher’s exact_ = 7.50*10^−3^, Odds ratio = 0.52). In addition, LD calculation suggested that in schizophrenic patients, stronger LD observed between STin2 and 5-HTTLPR in comparison with healthy controls (total: D′ = 0.57, LOD = 23.20, r^2^ = 0.14/cases: D′ = 0.62, LOD = 8.12, r^2^ = 0.16/controls: D′ = 0.53, LOD = 14.69, r^2^ = 0.13). These findings respectively showed in Tables 6 and 7. Besides, Fig. 1 shows S and STin2.12 alleles haplotype combination in cases and controls.

**Table 6:** 5-HTTLPR and STin2 risk alleles interaction.

5-HTTLPR	STin2		
	10/10	12/(10,12)	*P* value
**Cases**			
LL	5 (2%)	41 (17%)	0.03
S-	5 (2%)	187(79%)	
**Controls**			
LL	32 (9%)	68 (18%)	1.90*10^−9^
S-	20 (5%)	249 (68%)	
**Total**			
LL	38 (6%)	109 (17%)	3.55*10^−12^
S-	25 (4%)	439 (73%)

**Table 7:** Haplotype analysis of S/STin2.12.

Haplotype	Case	Control	*P* value	*P* value (total)
S-10	24 (5%)	67 (9%)	0.01	4.81*10^−3^
L-10	96 (20%)	177 (24%)	0.11	
S-12	242 (50%)	311 (42%)	5.97*10^−3^	
L-12	120 (25%)	185 (25%)	1.00

### Prediction of SCZ risk using 5-HTTLPR & STin2

After removal of confounding variables effects, logistic regression discriminated that STin2.12 significantly increased risk of SCZ (*p* value_adjusted_ = 4.17*10^−3^) but, failed to show such an effect in 5-HTTLPR (*p* value_adjusted_ = 0.09). Nevertheless,” S—“and “STin2.12—“together could be significantly considered as a predictor for risk of SCZ (*p* value_adjusted_ = 8.24*10^−3^). General linear model results are expressed in Tables 8 and 9.

**Table 8:** General linear model for risk of SCZ using 5-HTTLPR and STin2.

	Estimate	SE	t value	Adjusted *p* value
Intercept	0.94	0.10	9.43	< 0.01*10^−13^
5-HTTLPR	− 0.06	0.04	− 1.30	9.81*10^−1^
STin2	− 0.23	0.07	− 3.35	4.17*10^−3^
Sex	4.21*10^−3^	0.04	− 0.08	1
Age	2.51*10^−3^	1.94*10^−3^	− 1.30	1

**Table 9:** General linear model for risk of SCZ using 5-HTTLPR-STin2 interaction.

	Estimate	SE	t value	Adjusted *p* value
Intercept	0.78	0.08	9.35	< 0.08*10^−14^
5-HTTLPR-STin2	− 0.13	0.04	− 3.10	8.24*10^−3^
Sex	2.70*10^−3^	0.04	0.05	1
Age	2.51*10^−3^	1.94*10^−3^	− 1.30	0.70

## Discussion

In this study, we hypothesized that there might be a significant association between the incidence of SCZ and three well-known functional polymorphisms in serotonin reuptake pathway genes. We failed to highlight the association of *MAOA* uVNTR and 5-HTTLPR risk alleles in the pathology of SCZ, but we found that STin2.12 repeat apart or as a haplotype block with 5-HTTLPR “S” allele significantly associated with SCZ in Iranian population and S-12 haplotype significantly enhance the risk of SCZ occurrence. Moreover, our findings discriminated the protective role of S-10 haplotype in healthy individuals for the first time.

As a monoamine neurotransmitter, 5-HT is one of the substantial components of the neuromodulation system which implicates in plenty of psychiatric conditions. The main regulator of 5-HT is 5-HTT localized in presynaptic neurons in synaptic cleft^49^. In 2018, lung et al. supported the idea that *SLC6A4* mRNA level changes during treatment of patients with major depression and correlates with their responses to treatment^50^. Such observations could shed a light on the importance of *SLC6A4* mRNA level as an endophenotype for clinical conditions like depression.

On the other aspect, VNTRs are subclasses of mini-satellites frequently found in the non-coding region of gene-rich areas. It has been proven that in some circumstances, VNTRs have evolved as a binding site for transcription factors and in such a manner, they regulate the product level of genes responsible for^51^. In 2021, Bakhtiari et al. discovered that 49% of expression-mediated VNTRs, have a strong causal effect on their nearby genes^52^. So, it can be concluded that *SLC6A4* functional polymorphisms such as 5-HTTLPR and STin2 might affect mood changes through alteration in the *SLC6A4* mRNA level. This idea was justified when other studies revealed the association of other *SLC6A4* variants with comorbid conditions including depressive-like symptoms and suicidal attempts in patients with SCZ^53,54^.

According to the previous studies, association investigation of *SLC6A4* polymorphisms independently may result paradoxically and it should be better to explore them as a haplotype. Several studies have attempted to look at this issue from such a perspective. Association of 5-HTTLPR/5-HTT rs25531 (L_A_) haplotype and 5-HTTLPR/STin2 (L10) haplotype with attempt to suicide in schizophrenic patients^55^ or STin2/HTT-3'UTR-SNP (T-10) association with autism in West Bengal population (cases: D′ = 0.82, r^2^ = 0.34/controls = D′ = 0.27, r^2^ = 0.05)^41^ are examples of such an approach.

However, it seems that among all *SLC6A4* haplotype blocks, the 5-HTTLPR/STin2 haplotype is more remarkable due to the effect of both loci on *SLC6A4* expression in an almost similar way. New findings suggest that in rat cortical cell culture, both 5-HTTLPR and STin2 prepare binding sites for CCCTC-binding factor (CTCF) which was previously known to only have a binding site on STin2. These new results suggest that in the case of S/STin2.12, the binding of CTCF makes more significant changes in contrast to other combinations^56^.

Association of S/STin2.10 haplotype with response to lithium prophylaxis treatment in bipolar patients and L/STin2.10 haplotype with suicidal attempt among Russian females are a few examples of studies focused on 5-HTTLPR/STin2 haplotype^57,58^. But, in the case of SCZ, the majority of explorations show the association of mentioned haplotype in combination with other polymorphisms. For instance, in 2008, one study revealed that 5-HTTLPR/STin2/rs104701/rs1042173 in the form of L/STin2.10/G/T associated with SCZ but not major depressive disorder^59^.

As mentioned above, up to now, no study has investigated the association 5-HTTLPR/STin2 haplotype with SCZ without considering any specific traits. Thus, we observed that 5-HTTLPR S allele and STin2.12 repeats are significantly more frequent among subjects not only separately but also in one haplotype block. Besides, it is taken from the general linear model that STin2.12 repeats carriers are more at the risk of SCZ. But, being a 5-HTTLPR S allele carrier alone does not affect SCZ risk. Whereas, carrying both S and STin2.12 does increase the risk of SCZ.

The origin of this observation might lie within the effect of the 5-HTT level and its variants on interface traits and brain functions. For example, in 2012, Zilles et al. showed that “L” homozygosity in 5-HTTLPR is associated with better performance in verbal working memory tasks which its impairment significantly has been observed with SCZ^60^. On the other hand, children and adolescents with homozygosity in the “S” allele of 5-HTTLPR have weaker connectivity in the superior medial frontal cortex. According to the previous findings, this brain region has shown higher metabolic rate during resting state fluorodeoxyglucose positron emission tomography (FDG-PET) imaging in schizophrenic patients who experienced auditory verbal hallucinations^61^. On the other aspect, among different brain regions, the amygdala is a particular target of 5-HTT. the hyper-methylation of *SLC6A4* in promoter—which significantly observed more in “L” carriers of 5-HTTLPR- is associated with reduction of left amygdala volume in males with SCZ^62^. It should be considered that previous evidence suggests the amygdala volume reduction makes individuals more exposed to SCZ^63^. Also, these epigenetic remodeling might be the further result of CTCF binding to 5-HTTLPR and STin2 VNTRs^56,64^.

There were several limitations to this study. First, we only assessed schizophrenic patients who had been referred to Roozbeh hospital and had Iranian nationality. Thus, it is hard to generalize these results and this should be confirmed in other populations. Besides, with increasing sample size, the results are more universal and could be led to the emergence of a new biomarker for the prognosis of SCZ. Moreover, it should be noted that all the results obtained from MAOA uVNTR risk allele are fragile because they are performed only on a small population of females and need to be studied in a larger population.

In conclusion, in this study, we zoomed on to confirm the association of *MAOA* uVNTR, 5-HTTLPR, and STin2 functional polymorphisms with SCZ in the Iranian population and for the first time, we formed a haplotype block between 5-HTTLPR and STin2 in schizophrenic and healthy individuals. There was no significant association between being exposed to SCZ and *MAOA* uVNTR specific allele. However, 5-HTTLPR and STin2, S, and 12 alleles either apart or in one haplotype block increase the risk of SCZ.

## References

[1] Kahn RS (2015). Schizophrenia. Nat. Rev. Dis. Primers.

[2] Lichtenstein P (2009). Common genetic determinants of schizophrenia and bipolar disorder in Swedish families: A population-based study. Lancet.

[3] Marsden CA (2006). Dopamine: The rewarding years. Br. J. Pharmacol..

[4] Hamon M (1990). The main features of central 5-HT1 receptors. Neuropsychopharmacology.

[5] Xu FL, Wang BJ, Yao J (2019). Association between the SLC6A4 gene and schizophrenia: An updated meta-analysis. Neuropsychiatr. Dis. Treat..

[6] Lesch KP (1996). Association of anxiety-related traits with a polymorphism in the serotonin transporter gene regulatory region. Science (New York N. Y.).

[7] Kobiella A (2011). How the serotonin transporter 5-HTTLPR polymorphism influences amygdala function: The roles of in vivo serotonin transporter expression and amygdala structure. Transl. Psychiatry.

[8] Culej J, Štefanović M, Ćelap I, Nikolac N, Karlović D (2015). Serotonin transporter polymorphism (5-HTTLPR) in Croatian population. Mol. Biol. Rep..

[9] Sabol SZ, Hu S, Hamer D (1998). A functional polymorphism in the monoamine oxidase A gene promoter. Hum. Genet..

[10] Lan NC (1989). Human monoamine oxidase A and B genes map to Xp 11.23 and are deleted in a patient with Norrie disease. Genomics.

[11] Mohammadi A, Rashidi E, Amooeian VG (2018). Brain, blood, cerebrospinal fluid, and serum biomarkers in schizophrenia. Psychiatry Res..

[12] Kim JH (2017). Altered interregional correlations between serotonin transporter availability and cerebral glucose metabolism in schizophrenia: A high-resolution PET study using [(11)C]DASB and [(18)F]FDG. Schizophr. Res..

[13] Chen ZY, Powell JF, Hsu YP, Breakefield XO, Craig IW (1992). Organization of the human monoamine oxidase genes and long-range physical mapping around them. Genomics.

[14] Babushkina NP, Kucher AN (2011). Functional role of VNTR polymorphism of human genes. Russ. J. Genet..

[15] Eslami Rasekh M, Hernández Y, Drinan SD, Fuxman Bass JI, Benson G (2021). Genome-wide characterization of human minisatellite VNTRs: Population-specific alleles and gene expression differences. Nucl. Acids Res..

[16] Vijayan NN (2009). Evidence of association of serotonin transporter gene polymorphisms with schizophrenia in a South Indian population. J. Hum. Genet..

[17] Sugawara H (2011). Hypermethylation of serotonin transporter gene in bipolar disorder detected by epigenome analysis of discordant monozygotic twins. Transl. Psychiatry.

[18] Hariri AR (2002). Serotonin transporter genetic variation and the response of the human amygdala. Science (New York N. Y.).

[19] Hu XZ (2006). Serotonin transporter promoter gain-of-function genotypes are linked to obsessive-compulsive disorder. Am. J. Hum. Genet..

[20] Wendland JR (2006). Differential functional variability of serotonin transporter and monoamine oxidase A genes in macaque species displaying contrasting levels of aggression-related behavior. Behav. Genet..

[21] Heils A (1996). Allelic variation of human serotonin transporter gene expression. J. Neurochem..

[22] Heils A (1995). Functional promoter and polyadenylation site mapping of the human serotonin (5-HT) transporter gene. J. Neural Transm. Gen. Sect..

[23] Xu F-L, Wang B-J, Yao J (2018). Association between the SLC6A4 gene and schizophrenia: An updated meta-analysis. Neuropsychiatr. Dis. Treat..

[24] Semwal P (2001). Family-based association studies of monoaminergic gene polymorphisms among North Indians with schizophrenia. Mol. Psychiatry.

[25] Golimbet V, Korovaitseva G, Lezheiko T, Abramova LI, Kaleda VG (2017). The serotonin transporter gene 5-HTTLPR polymorphism is associated with affective psychoses but not with schizophrenia: A large-scale study in the Russian population. J. Affect. Disord..

[26] Xu-xiu J (2016). Study on the correlation between the polymorphism in promoter region of 5-hydroxytryptamine transporter gene and schizophrenia in Chinese Han population. J. Shanghai Jiaotong Univ. (Med. Sci.).

[27] Lesch KP (1994). Organization of the human serotonin transporter gene. J. Neural Transm. Gen. Sect..

[28] Kaiser R (2001). Serotonin transporter polymorphisms: No association with response to antipsychotic treatment, but associations with the schizoparanoid and residual subtypes of schizophrenia. Mol. Psychiatry.

[29] Klenova E (2004). YB-1 and CTCF differentially regulate the 5-HTT polymorphic intron 2 enhancer which predisposes to a variety of neurological disorders. J. Neurosci. Off. J. Soc. Neurosci..

[30] MacKenzie A, Quinn J (1999). A serotonin transporter gene intron 2 polymorphic region, correlated with affective disorders, has allele-dependent differential enhancer-like properties in the mouse embryo. Proc. Natl. Acad. Sci. U. S. A..

[31] Gomes CKF (2018). Association analysis of SLC6A4 and HTR2A genes with obsessive–compulsive disorder: Influence of the STin2 polymorphism. Compr. Psychiatry.

[32] Kazantseva AV (2008). Polymorphisms of the serotonin transporter gene (5-HTTLPR, A/G SNP in 5-HTTLPR, and STin2 VNTR) and their relation to personality traits in healthy individuals from Russia. Psychiatr. Genet..

[33] Fresan A (2007). Association study of MAO-A and DRD4 genes in schizophrenic patients with aggressive behavior. Neuropsychobiology.

[34] Zammit S (2004). Polymorphisms in the MAOA, MAOB, and COMT genes and aggressive behavior in schizophrenia. Am. J. Med. Genet. Part B Neuropsychiatr. Genet. Off. Publ. Int. Soc. Psychiatric Genet..

[35] Bortolato M, Shih JC (2011). Behavioral outcomes of monoamine oxidase deficiency: Preclinical and clinical evidence. Int. Rev. Neurobiol..

[36] Brunner HG, Nelen M, Breakefield XO, Ropers HH, van Oost BA (1993). Abnormal behavior associated with a point mutation in the structural gene for monoamine oxidase A. Science (New York N. Y.).

[37] Manca M (2018). The regulation of monoamine oxidase A gene expression by distinct variable number tandem repeats. J. Mol. Neurosci..

[38] Camarena B (2012). Monoamine oxidase a and B gene polymorphisms and negative and positive symptoms in schizophrenia. ISRN Psychiatry.

[39] Sun Y (2012). Study of a possible role of the monoamine oxidase A (MAOA) gene in paranoid schizophrenia among a Chinese population. Am. J. Med. Genet. Part B Neuropsychiatr. Genet. Off. Publ. Int. Soc. Psychiatric Genet..

[40] Culej J, Nikolac Gabaj N, Štefanović M, Karlović D (2020). Prediction of schizophrenia using MAOA-uVNTR polymorphism: A case–control study. Indian J. Psychiatry.

[41] Guhathakurta S (2008). Population-based association study and contrasting linkage disequilibrium pattern reveal genetic association of SLC6A4 with autism in the Indian population from West Bengal. Brain Res..

[42] Alizadeh F, Bozorgmehr A, Tavakkoly-Bazzaz J, Ohadi M (2018). Skewing of the genetic architecture at the ZMYM3 human-specific 5′ UTR short tandem repeat in schizophrenia. Mol. Genet. Genom. MGG.

[43] Gonda X (2006). The 5HTTLPR polymorphism of the serotonin transporter gene is associated with affective temperaments as measured by TEMPS-A. J. Affect. Disord..

[44] Samochowiec A (2015). Monoamine oxidase a promoter variable number of tandem repeats (MAOA-uVNTR) in alcoholics according to Lesch typology. Int. J. Environ. Res. Public Health.

[45] de Castro MRP (2014). SLC6A4 STin2 VNTR genetic polymorphism is associated with tobacco use disorder, but not with successful smoking cessation or smoking characteristics: A case control study. BMC Genet..

[46] Barrett JC, Fry B, Maller J, Daly MJ (2005). Haploview: Analysis and visualization of LD and haplotype maps. Bioinformatics (Oxford, England).

[47] De Neve J-E (2011). Functional polymorphism (5-HTTLPR) in the serotonin transporter gene is associated with subjective well-being: Evidence from a US nationally representative sample. J. Hum. Genet..

[48] Fan JB, Sklar P (2005). Meta-analysis reveals association between serotonin transporter gene STin2 VNTR polymorphism and schizophrenia. Mol. Psychiatry.

[49] Haddley K, Bubb VJ, Breen G, Parades-Esquivel UM, Quinn JP (2012). Behavioural genetics of the serotonin transporter. Curr. Top. Behav. Neurosci..

[50] Kao WT, Chang CL, Lung FW (2018). 5-HTT mRNA level as a potential biomarker of treatment response in patients with major depression in a clinical trial. J. Affect. Disord..

[51] Søeby K, Larsen SA, Olsen L, Rasmussen HB, Werge T (2005). Serotonin transporter: Evolution and impact of polymorphic transcriptional regulation. Am. J. Med. Genet. Part B Neuropsychiatr. Genet. Off. Publ. Int. Soc. Psychiatric Genet..

[52] Bakhtiari M (2021). Variable number tandem repeats mediate the expression of proximal genes. Nat. Commun..

[53] Peitl V, Štefanović M, Karlović D (2017). Depressive symptoms in schizophrenia and dopamine and serotonin gene polymorphisms. Prog. Neuropsychopharmacol. Biol. Psychiatry.

[54] Hung CF (2011). Association between suicide attempt and a tri-allelic functional polymorphism in serotonin transporter gene promoter in Chinese patients with schizophrenia. Neurosci. Lett..

[55] Božina N (2012). Suicide ideators and attempters with schizophrenia—The role of 5-HTTLPR, rs25531, and 5-HTT VNTR Intron 2 variants. J. Psychiatr. Res..

[56] Ali FR (2010). Combinatorial interaction between two human serotonin transporter gene variable number tandem repeats and their regulation by CTCF. J. Neurochem..

[57] Tharoor H, Kotambail A, Jain S, Sharma PS, Satyamoorthy K (2013). Study of the association of serotonin transporter triallelic 5-HTTLPR and STin2 VNTR polymorphisms with lithium prophylaxis response in bipolar disorder. Psychiatr. Genet..

[58] Gaysina D, Zainullina A, Gabdulhakov R, Khusnutdinova E (2006). The serotonin transporter gene: Polymorphism and haplotype analysis in Russian suicide attempters. Neuropsychobiology.

[59] Zaboli G (2008). Haplotype analysis confirms association of the serotonin transporter (5-HTT) gene with schizophrenia but not with major depression. Am. J. Med. Genet. Part B Neuropsychiatr. Genet. Off. Publ. Int. Soc. Psychiatric Genet..

[60] Conklin HM, Curtis CE, Katsanis J, Iacono WG (2000). Verbal working memory impairment in schizophrenia patients and their first-degree relatives: Evidence from the digit span task. Am. J. Psychiatry.

[61] Horga G (2011). Differential brain glucose metabolic patterns in antipsychotic-naïve first-episode schizophrenia with and without auditory verbal hallucinations. J. Psychiatry Neurosci. JPN.

[62] Ikegame T (2020). Promoter activity-based case–control association study on SLC6A4 highlighting hypermethylation and altered amygdala volume in male patients with schizophrenia. Schizophr. Bull..

[63] Rich AM (2016). Amygdala volume is reduced in early course schizophrenia. Psychiatry Res. Neuroimaging.

[64] Ohlsson R, Renkawitz R, Lobanenkov V (2001). CTCF is a uniquely versatile transcription regulator linked to epigenetics and disease. Trends Genet. TIG.

